# Yeast-Mediated Plastic Biodegradation

**DOI:** 10.3390/ijms27093939

**Published:** 2026-04-28

**Authors:** Xin-Yue Yang, Lin-Bei Xie, Zhong-Wei Zhang, Shu Yuan

**Affiliations:** International Science and Technology Cooperation Base for Efficient Utilization of Nutrient Resources and Fertilizer Innovation, College of Resources, Sichuan Agricultural University, Chengdu 611130, China; yang16970319@163.com (X.-Y.Y.); kkskado@163.com (L.-B.X.); zzwzhang@126.com (Z.-W.Z.)

**Keywords:** biodeterioration, depolymerization, mineralization, plastics, yeast

## Abstract

Plastic pollution is a global environmental crisis, and microbial degradation represents a promising remediation strategy. While bacteria have been widely studied, yeasts offer unique advantages for plastic degradation due to their metabolic versatility, stress tolerance, and enzymatic capabilities. However, plastic degradative yeasts have not been reviewed comprehensively. Although several yeasts capable of degrading polyethylene terephthalate (PET) or polyethylene (PE) have been reported (e.g., *Moesziomyces antarcticus*, *Candida tropicalis*, *Yarrowia lipolytica* and *Rhodotorula mucilaginosa*), degraders of other plastic types are less studied. Although some yeasts can assimilate carbon from plastics, the diversity of yeasts capable of participating in plastic mineralization remains vastly underexplored. In recent years, yeast cell surface display systems for bacterial PETase and fungal cutinase have been developed, demonstrating promising PET degradation efficiency. However, PETase is feedback-inhibited by the intermediate product mono(2-hydroxyethyl)terephthalate (MHET). Systems synergizing PETase with MHETase have shown superior stability during long-term PET degradation and enable large-scale depolymerization of PET waste. For high-crystallinity PET, fungal hydrophobins can be used to modify the surface hydrophobicity of PETase-displaying yeast cells, facilitating their attachment to hydrophobic PET surfaces and ultimately enhancing the degradation efficiency of the whole-cell biocatalyst. Limitations of current research and future directions are also discussed.

## 1. Introduction

Due to their advantageous properties—including flexibility, light weight, and corrosion resistance—plastic polymers are both highly desirable and easy to manufacture. These materials are created via polymerization, a process that can involve chemical additives [1]. Their environmental persistence stems from extensive use and improper disposal. Once in the environment, plastics fragment into particles categorized by size: mega-, meso-, micro-, and nano-plastics. These particles are now ubiquitous, found in water resources, sediments, and various containers. Secondary microplastics (MPs) and nano-plastics (NPs) are generated through long-term UV exposure, physical weathering, and physiochemical degradation. The scale of contamination from common thermoplastics like polyethylene (PE), high-density PE (HDPE), low-density PE (LDPE), polypropylene (PP), and polyvinyl chloride (PVC) is now comparable to that of nanoparticles [2].

A major environmental and ecological issue stems from plastic waste, which results from high production volumes and incorrect disposal practices. Significant CO_2_ emissions are released throughout the plastic lifecycle, and poorly managed waste contaminates ecosystems as litter or in landfills. An additional threat comes from microplastics, which impact terrestrial and marine life and pose risks to food security and public health [3,4,5]. The most widespread plastic in consumer items is polyethylene terephthalate (PET), making up about 12% of global solid waste. However, established physical and chemical recycling techniques are problematic due to their high economic and energy costs, as well as their potential to cause secondary pollution [6]. Biological recycling has thus emerged as an eco-friendly and energy-efficient pathway for processing PET, offering advantages in simplicity and sustainability [7].

Micro- and nano-plastics containing plasticizers are highly vulnerable to microbial degradation under diverse environmental conditions. Bacteria and fungi can break down plasticizers such as organic acid esters (e.g., dioctyl phthalate [DOP] and dioctyl adipate [DOA]), altering the stability of polymer nanoparticles. The microbial breakdown of these additives results in structural weakening, making the material brittle, prone to shrinkage, and ultimately causing failure [8]. In recent years, researchers have discovered multiple enzymes capable of hydrolyzing polyethylene terephthalate (PET), such as lipases, esterases, carboxylesterases, and cutinases [9]. However, significant enzymatic activity typically requires elevated temperatures (close to or above PET’s glass transition temperature of about 70 °C) as well as highly purified substrates [10]. Additionally, most known PET-degrading enzymes exhibit limited efficiency at moderate temperatures, hindering their effectiveness in natural or microbial degradation processes. This is concerning, given that around 40% of plastic waste remains uncollected, contributing to environmental pollution.

Plastic pollution represents a worldwide environmental challenge, for which microbial degradation offers a potential solution. So far, various plastic-degrading bacteria are being isolated from soil and marine environments, such as *Piscinibacter sakaiensis*, *Pseudomonas* spp., *Bacillus* spp., *Alcanivoras* spp. and Actinomycetes [10,11]. Although these bacterial species have been the focus of extensive research, yeasts present distinct benefits for breaking down plastics. Yeasts are single-celled fungi that reproduce primarily by budding or fission and are characterized by their ability to metabolize organic compounds under both aerobic and anaerobic conditions. They have garnered increasing attention as promising agents for plastic biodegradation due to their rapid growth rates, high metabolic versatility, and genetic tractability [12]. Compared to filamentous fungi, yeasts offer several distinct advantages for this application. First, their unicellular nature allows for more efficient and homogeneous contact with plastic surfaces, facilitating enzymatic attack. Yeasts, including genera like *Candida* and *Rhodotorula*, can adhere strongly to plastic surfaces, particularly microplastics (MPs), by forming robust biofilms. This adhesion is facilitated by their hydrophobic cell walls and enhances direct enzymatic action on the plastic [13]. Second, yeasts generally exhibit greater tolerance to environmental stressors such as high osmotic pressure, acidic pH, and variable temperatures, making them more robust for industrial-scale bioprocessing. *Candida tropicalis*, for instance, is noted for its proficiency in degrading polycyclic aromatic hydrocarbons and its notable resistance to environmental stress, making it a promising candidate for managing pollutants like PET waste [14,15]. Third, the well-established genetic engineering tools available for model yeasts like Saccharomyces cerevisiae enable straightforward modification to enhance plastic-degrading enzyme expression and activity [16,17]. Finally, yeasts can be cultivated in simple, low-cost media and are less prone to the morphological complications and sporulation issues that often hinder filamentous fungi in bioreactor systems [12]. These combined characteristics position yeasts as highly attractive candidates for developing sustainable biological strategies to address plastic pollution.

A number of yeast species capable of breaking down PET or PE have been documented, yet microorganisms that can degrade other categories of plastics remain comparatively unexamined. While certain yeasts are able to metabolize carbon derived from plastic materials, the full range of yeast species involved in plastic mineralization has yet to be thoroughly characterized. In recent years, researchers have constructed yeast cell surface display systems expressing bacterial PETase and fungal cutinase, which have shown encouraging activity in PET degradation. Nevertheless, PETase activity is subject to feedback inhibition caused by intermediate products. Furthermore, when applied to high-crystallinity PET, these yeast surface display systems continue to demonstrate limited efficiency. This review addresses the current limitations in the field and outlines future directions for advancing yeast-mediated plastic biodegradation.

## 2. Yeast-Mediated Plastic Mineralization and Depolymerization

Once plastic components have been successfully transported into the cell, a cascade of enzymatic reactions occurs, ultimately breaking down the plastic materials into oxidized end products such as H_2_O, N_2_, CH_4_, and CO_2_. This mineralization process can take place under either aerobic or anaerobic conditions. A range of enzyme activities—including peroxidases, lipases, esterases, cutinases, and laccases—is necessary to drive the complete mineralization pathway. Ultimately, all biodegradable compounds are utilized by microorganisms, and the entire carbon content is released as CO_2_ [18,19]. But the mineralization only occurs to a limited degree. In one study, Gerritse et al. [20] examined the breakdown of both conventional thermoplastic and compostable plastic materials within a controlled seawater laboratory microcosm. The surfaces of these plastics became colonized by microbial biofilms consisting primarily of Cyanobacteria, Proteobacteria, Planctomycetes, and Bacteroidetes, and this biofilm growth led to the sinking of certain polyethylene items. Annual mass loss for PE, polystyrene (PS), and PP was no more than 1%, whereas latex, PET, and polyurethane (PU) exhibited rates between 3% and 5%.

Comparatively, yeasts show higher plastic mineralization efficiency. Two novel yeast strains, identified as *Exophiala* sp. NS-7 and *Rhodotorula* sp. NS-12, were isolated for their ability to degrade polyester-polyether urethanes (PPU). The characterization results indicated that *Exophiala* sp. NS-7 tested positive for esterase, protease, and urease activity, while *Rhodotorula* sp. NS-12 produced esterase and urease. When provided with Impranil^®^ (a commercial PPU) as a sole carbon source, both strains demonstrated degradation, achieving their highest growth rates after 4–6 days and 8–12 days, respectively. Scanning electron microscope (SEM) analysis provided visual evidence of this degradation, revealing numerous pits and holes in the treated PPU films. Furthermore, a Sturm test confirmed that both isolates could mineralize PPU into carbon dioxide, and the Fourier-transform infrared spectroscopy (FTIR) showed significant reductions in key absorption bands corresponding to N-H stretching, C-H stretching, C=O stretching, and N-H/C=O bending in the polymer’s molecular structure [21].

A separate investigation employed a stable isotope tracing assay with ^13^C-labeled PE to quantify degradation by the marine yeast *Rhodotorula mucilaginosa*, measuring a rate of 3.8% per year. This work highlighted the potential of *R. mucilaginosa* to both mineralize and assimilate carbon from plastics, suggesting that fungal degradation could represent a significant sink for polyethylene litter in marine ecosystems [22].

In another study, an esterase produced by the phyllosphere yeast *Moesziomyces antarcticus* (*Candida antarctica*) was shown to enhance the degradation and mineralization of poly(butylene succinate-co-adipate) (PBSA) film in soil. The research found that *M. antarcticus* could functionally substitute for esterases from initial degraders and stimulate subsequent esterase production from indigenous soil microbes. Consequently, PBSA film pretreated with the *M. antarcticus* esterase degraded rapidly and was substantially mineralized within 55 days, as evidenced by high carbon dioxide production [23] (Table 1).

Polyester plastics are formed from long-chain macromolecules whose high molecular weight prevents direct uptake by microbial cells, necessitating that their degradation occurs via extracellular processes [47]. Research has shown that a mono(hydroxyethyl terephthalate)-converting lipase from *M. antarcticus* can catalyze the breakdown of post-consumer PET packaging into its constituent monomers [24].

In a study by El-Dash et al. [40], 18 fungi capable of biodegrading plastic were isolated from landfill soil samples. Among these, only 10 isolates demonstrated the ability to degrade PVC polymer, with four exhibiting notable depolymerase activity. Molecular identification classified one of these active isolates as belonging to the yeast genus *Malassezia*.

The yeast *Sakaguchia* sp. BIT-D3 secretes extracellular enzymes that cleave the ester bonds in polyester. To pinpoint the specific enzymes responsible, Huang et al. [31] performed integrated genomic and transcriptomic analyses on this strain, which was isolated from the gut of plastic-eating mealworms. Their work led to the identification of two novel cutinases, SiCut1 and SiCut2. Despite sharing less than 25% sequence identity with previously known cutinases, both enzymes demonstrated significant depolymerization activity against multiple polyester plastics, including polycaprolactone (PCL) film, polybutylene succinate (PBS) film, and PPU foam [31] (Table 1).

## 3. Plastic Biodeterioration Yeasts

**Figure 1:**
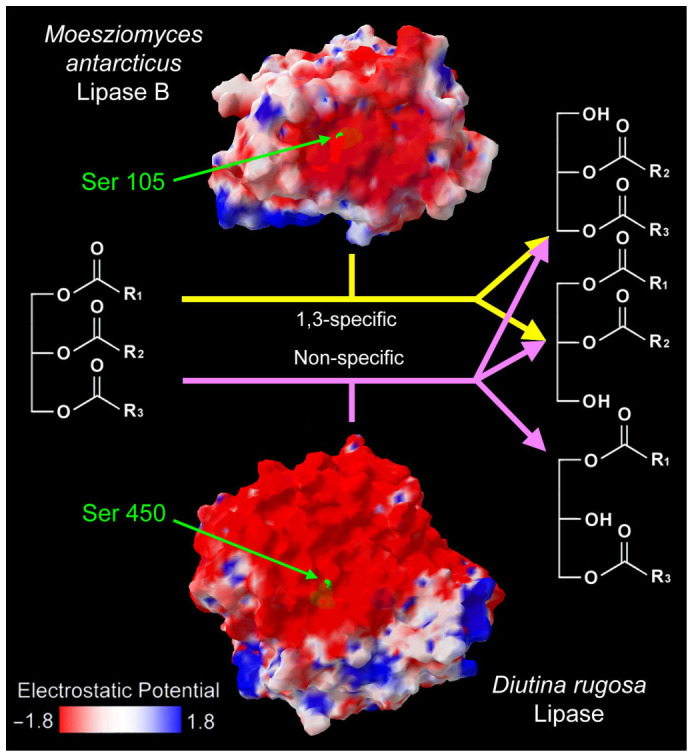
Electrostatic potential of two representative yeast lipases. Catalytic serine on the substrate-binding area is marked with a green color. The molecular surface and the electrostatic potential were computed by the Swiss-PdbViewer v4.1.0 software. The PDB templates for *M. antarcticus* lipase B and *D. rugosa* lipase were 1TCA and 1LPM respectively. The red-to-blue color gradient on the molecular surface indicates the electrostatic potential (red: −1.8; blue: 1.8).

Fungi and other microorganisms can break down plastics using specialized enzymes, [16,17]. The ester bonds connecting plastic’s monomeric units are vulnerable to hydrolysis by yeast enzymes such as esterases, lipases, and cutinases [10,16,17]. Specifically, esterases target these short-chain acyl ester bonds and can alter the surface properties of plastics [48]. Lipases, known for their interfacial activation, contribute to plastic fabric hydrolysis by increasing material wettability. Cutinases, recognized as particularly effective lipolytic enzymes for plastic degradation, are notably produced by various fungal species [16,17]. For example, plastics may bind *Moesziomyces antarcticus* lipase B catalytic serine 105; while they may bind *Diutina rugosa* lipase at the residue Ser 450. On the other hand, different yeast lipases have different regioselectivities (*M. antarcticus* lipase B is a 1,3 specific enzyme for triacylglycerol substrates; while *D. rugosa* lipase is non-specific) [49] (Figure 1).

### 3.1. PET Biodeterioration Yeasts

**Figure 2:**
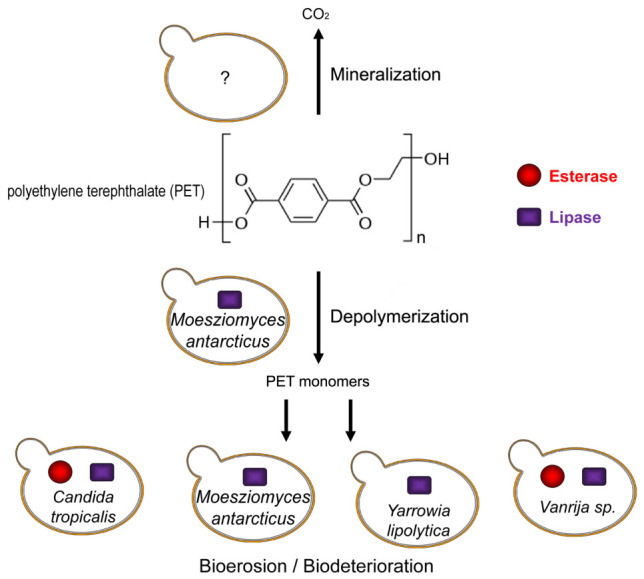
PET degradative yeasts. *Moesziomyces antarcticus* catalyzes the conversion of PET into monomers. *Moesziomyces antarcticus*, *Candida tropicalis*, *Yarrowia lipolytica* and *Vanrija* sp. show PET-degrading activities with their lipase and esterase. Nevertheless, yeasts specific to PET mineralization have not been identified yet.

Research indicates that certain fungi metabolize PET into simpler compounds like bis(2-hydroxyethyl)terephthalate (BHET) and mono(2-hydroxyethyl)terephthalate (MHET) [16,17]. And yeast-derived lipases and esterases play predominant roles in PET hydrolysis. For instance, enzymes from *M. antarcticus* and *Y. lipolytica* have been shown to break down PET into terephthalic acid (TPA) [24,25,26]. Furthermore, *C. tropicalis* has been reported to degrade 50% of PET-based microplastics over a 30-day period [27]. A newly isolated strain, *Vanrija* sp. SlgEBL5, also exhibits lipase and esterase activity conducive to PET microplastic degradation [28], as detailed in Table 1 and Figure 2.

### 3.2. PE Biodeterioration Yeasts

Microbial consortia capable of degrading PE were cultivated through sequential enrichment in a medium containing linear low-density polyethylene (LLDPE), provided as either film or powder, as the sole carbon source. From one such consortium, the yeast *Debaryomyces hansenii* RELF8 was isolated and identified based on its distinct polyethylene-degrading enzymatic profile [29]. Furthermore, a recent screening of six yeast strains from marine plastic debris in East Java, Indonesia, identified *Meyerozyma carpophila* M6.0.2 as the most effective. This strain achieved a 0.5% degradation of polyethylene in liquid culture over ten days. Enzymatic analysis revealed the presence of several PE-modifying enzymes in this species, including laccase, glutathione peroxidase, non-heme chloroperoxidase, cytochrome P450, esterase, and lipase [30] (Table 1).

### 3.3. PU Biodeterioration Yeasts

In a study by Gautam et al. [32], the enzymatic degradation of commercially available solid PU was investigated using *Diutina rugosa* lipase (EC 3.1.1.3) in an aqueous medium, with the process conditions varied to assess the effects of temperature, pH, and enzyme and substrate concentrations. In related research, Gallorini et al. [33] demonstrated that after subjecting PU to hydrothermal liquefaction with either ultrapure water or a basic potassium hydroxide (KOH) catalyst, the resulting organic fraction could be further digested using a lipase also derived from *D. rugosa*.

Evidence of yeast-mediated PU deterioration was also provided by Zafar et al. [34], who observed significant physical deterioration of polyester PU coupons, which was associated with extensive yeast colonization. Pyrosequencing analysis identified *Candida ethanolica* as the most dominant fungus on the surfaces of these coupons at both 45 °C and 50 °C.

More recently, a black yeast designated as *Hortaea werneckii* M-3, which was isolated from marine sediment in the West Pacific, was shown to utilize PU as a sole carbon source, with genomic analysis confirming the presence of genes encoding potential PU-degrading enzymes such as cutinase and urease [35].

Further demonstrating the diversity of plastic-degrading yeasts, a 2024 study by Kumar et al. [36] isolated yeast species including *Candida*, *Meyerozyma*, *Rhodotorula*, *Malassezia*, and *Rhodosporidiobolus* from various depths of a plastic-laden landfill. These isolates were respectively reported to exhibit degradative activity against PU, PE, non-UV and UV-treated PE, PVC, and PET (Table 1).

### 3.4. PP Biodeterioration Yeasts

According to Mihreteab et al. [50], a process for upcycling PP was established by integrating pyrolysis with subsequent bioconversion using the oleaginous yeast *Yarrowia lipolytica*. Subsequent research by the same group [37] utilized virgin PP to optimize key parameters including pH, inoculum density, carbon-to-nitrogen (C/N) ratio, and osmolarity. This optimization resulted in a nearly four-fold increase in fatty acid titer, achieving 1.9 g per liter, with a cellular fatty acid content of 41 percent, which was the highest content reported to date for yeast bioconversion of plastic to lipids.

In a screening study for environmental yeast isolates capable of degrading plastic polymers, Černoša et al. [38] employed a combination of SEM, FTIR, and Raman spectroscopy. Their research found that when cultivated in a minimal medium lacking a carbon source, certain strains exhibited a significant increase in growth upon the addition of a pure plastic polymer. Specifically, *Rhodotorula dairenensis* EXF-13500 showed enhanced activity with PP, an unidentified *Rhodotorula* species (EXF-10630) with low-density PE, and *Wickerhamomyces anomalus* EXF-6848 with PET.

Recent work by Weng et al. [39] confirmed that plastic degradation in *Zophobas atratus* superworms is mediated by their gut microbiota. To investigate if the type of plastic polymer influences both biodegradation and the gut microbial community, the researchers fed the superworms foams representing different polymer classes: PP for polyolefins, PU for polyesters, and ethylene vinyl acetate (EVA) for copolymers. The study revealed that, compared to bran-fed control groups, all plastic-fed groups experienced similar shifts in their gut fungal communities, characterized by an increase in the dominant abundance of the genus *Rhodotorula*. This increase in *Rhodotorula* abundance followed a trend, rising in the order from polyolefin, to polyester, and then to copolymer (Table 1).

### 3.5. Other Plastic Degradative Yeasts

While PET-degrading yeasts have been extensively researched, other plastics and the yeasts capable of degrading them have received comparatively less scientific attention [51]. For instance, the yeast-like fungi *Buckleyzyma aurantiaca* and *Kluyveromyces* spp. have demonstrated an ability to degrade PVC, though the specific enzymes responsible were not characterized in that study [41]. Similarly, *D. rugosa* has been reported to degrade a poly(butylene succinate-co-hexamethylene succinate) copolymer [P(BS-co-HS)] [42]. Research has identified a cutinase from *Cryptococcus* sp. strain S-2 that is effective against several polymers, including polylactic acid (PLA), polybutylene succinate (PBS), poly(epsilon-caprolactone) (PCL), and poly(3-hydroxybutyrate) (PHB) [43]. A lipase from a *Cryptococcus* sp. yeast was also shown to hydrolyze PBS and polybutylene succinate-co-adipate (PBSA) [44]. In the case of PCL, the wild-type *Y. lipolytica* strain AJD showed only a 1% weight loss over 144 h [45]. However, as will be discussed, engineered strains of *Y. lipolytica* can achieve dramatically higher degradation efficiencies exceeding 90% [45]. A lipase from *Candida* sp. can degrade low molecular weight PCL [46] (Table 1).

## 4. Chemicals Promote Yeast-Mediated Plastic Biodegradation

Research has shown that the biodegradation of PET microplastics can be enhanced through the addition of specific agents. For example, the biodegradation rate of PET by the yeast *Vanrija*sp. SlgEBL5 increased from 10% to 16.6% when the surfactant Tween 20 was added to lower surface tension [28].

Similarly, the solvent dimethyl sulfoxide (DMSO) was used to improve the depolymerization of post-consumer PET (PC-PET) by *Y. lipolytica*. A MIC test established 5% DMSO as the maximum non-toxic concentration for yeast cultivation. Notably, cell growth in a yeast nitrogen-based (YNB) medium with PC-PET was only observed when DMSO was present. With PC-PET as an additional carbon source, cell biomass increased by 40% in DMSO-supplemented cultures (reaching 10.7 g/L), and cell adhesion to PET surfaces improved by 20% [26].

In another study, supplementing the *Y. lipolytica* culture medium with olive oil resulted in higher extracellular lipase activity, more severe surface corrosion and a greater mass loss of PET film (53.05%) compared to the 44.74% loss observed in the unsupplemented control [52].

## 5. Yeast Cell Surface Display

### 5.1. Bacterial PETase and MHETase

A significant advance occurred in 2016 with the isolation of a PET hydrolase, termed PETase, from *Piscinibacter sakaiensis* [53]. This enzyme operates under ambient conditions and can act on highly crystalline plastics, though it suffers from instability, losing activity within 24 h even at 37 °C [54]. Research has since aimed to enhance ambient-temperature PET degradation and improve in situ strategies. Engineered variants like FAST-PETase [55] and DepoPETase [56] offer greater activity and stability, enabling the full depolymerization of untreated postconsumer PET at moderate temperatures. Similarly, HotPETase [57] shows high degradation efficiency on postconsumer PET at moderate and near-glass transition temperatures. Despite these advances, the use of free enzymes is limited by challenges such as high purification costs, low solubility and stability, and self-aggregation [58]. As an alternative, displaying PETase on microbial cell surfaces, such as that of *Escherichia coli* [58,59], has been explored, yielding systems with higher stability and simpler operation. However, even these engineered biocatalysts are often hindered from complete substrate decomposition by inhibitory intermediate products like MHET [60]. Furthermore, host robustness is critical for efficient degradation, as the process extends over several days and produces high concentrations of TPA and ethylene glycol (EG) [24].

Multi-enzyme display platforms (MEDPs) utilize scaffold proteins to anchor enzymes onto a cellular surface. This arrangement enables sequential reactions, where the product of one enzyme acts directly as the substrate for the next. By minimizing the distance substrates must diffuse between enzymes, this proximity significantly improves catalytic efficiency. For PET degradation, MEDPs offer a particularly advantageous strategy. They can circumvent the competitive inhibition caused by accumulating intermediate products, thereby boosting the efficacy of the biological recycling process [61]. A significant barrier to scaling this technology, however, is the current multi-step requirement for protein purification and subsequent assembly with catalytic materials [61]. Furthermore, displaying larger enzyme constructs, such as MHETase–PETase chimeras, has led to issues of suboptimal activity [62] or incomplete substrate conversion [63]. These challenges highlight a need for a more integrated MEDP methodology, including a rational design framework to optimally synergize PETase and MHETase activities for efficient PET depolymerization.

Optimizing the synergy between PETase and MHETase within MEDPs requires careful consideration of each component. The protein scaffold is a critical element, as it dictates the spatial arrangement of enzymes to facilitate cooperative catalysis, enhances structural and functional stability, and enables modular design for precise control. Protein–peptide systems, such as SpyTag–SpyCatcher, are promising scaffold candidates due to their small size and high binding specificity, which are advantageous for linking complex, high-molecular-weight proteins [64]. The selection of the host chassis cell is another vital factor, as different microbial systems offer distinct properties. For instance, yeast display systems are particularly well-suited for presenting complex, multi-protein assemblies compared to other platforms [64].

**Figure 3:**
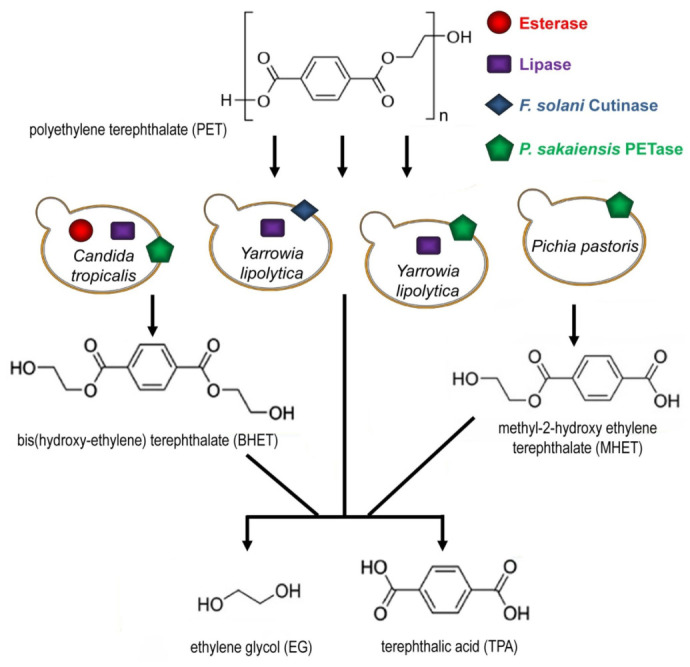
Cell surface displays in PET degradative yeasts. PET degradation results in two intermediates BHET and MHET and final products TPA and EG. Cell surface displays of *P. sakaiensis* PETase or *F. solani* cutinase in *Yarrowia lipolytica*, *Pichia pastoris* or *Candida tropicalis* along with yeast lipase and esterase show higher degradation efficiencies.

Zhang et al. [65] developed the Integrated Self-Assembling Multi-Enzyme Display Platform (ISA-MEDP) to enable the direct and efficient depolymerization of postconsumer PET (pc-PET). To achieve this, they engineered a novel surface display system in the non-conventional yeast *C. tropicalis*, facilitating the functional attachment of FAST-PETase and MHETase enzymes to the cell wall. By precisely controlling the co-display ratio of PETase and MHETase and optimizing the self-assembly sequence, the researchers constructed the ISA-MEDP. This platform demonstrated significantly enhanced degradation of untreated pc-PET with over 10% crystallinity at near-ambient temperatures. During a scaled batch process in a 5L bioreactor with pH as the sole controlled parameter, the system achieved complete depolymerization of 20 g of pc-PET powder within seven days. The study presents a novel and applicable system for enzyme co-display and assembly, offering a robust enzymatic catalyst with considerable potential for the large-scale recycling of PET in industrial applications [65] (Table 2 and Figure 3).

Zhang et al. [65] assessed the performance of their ISA-MEDP system against alternative approaches, including *E. coli*-based co-expression [61] and immobilized enzyme platforms such as calcium phosphate nanocrystals [70], MC@CaZn-MOF carriers [71], and iron oxide nanoparticles [72]. Key evaluation criteria included operational stability, degradation capacity, and tolerance to substrate crystallinity. The ISA-MEDP system provides a unique advantage by eliminating the need for enzyme purification, significantly streamlining the depolymerization process. Additionally, it demonstrates superior long-term stability and enables large-scale PET waste degradation, as demonstrated by the complete breakdown of 20g of PET powder in a 5L bioreactor at 45 °C within seven days [65].

Other yeast platforms have also been explored for PET degradation. For instance, an engineered *Y. lipolytica* Po1f strain producing PETase was shown to hydrolyze BHET and PET powder into TPA and EG [66]. Similarly, surface display of *Ps*PETase in a *Y. lipolytica* AJD strain achieved a 44.74% weight loss of PET film after 240 h at 28 °C [52].

### 5.2. Fungal Hydrophobin

Although the second catalytic step of PETase has been the primary focus of research, the role of the initial adsorption phase in determining degradation efficiency has received less attention [73]. Hydrophobins (HFBs), which are small secreted proteins from filamentous fungi, play key roles in developmental functions such as spore formation, aerial hyphae development, and fruiting body stability. A critical additional function of HFBs is their ability to mediate fungal adhesion to hydrophobic surfaces [74]. This adhesion is achieved through the self-assembly of HFBs at the interface between the fungal cell wall and a hydrophobic material [75], where a hydrophobic patch on the protein engages in robust hydrophobic interactions [76]. Their amphipathic nature allows hydrophobins to naturally alter cell surface hydrophobicity. Research indicates that hydrophobins can improve the adhesion of various enzymes, including those that degrade PET, to different materials in laboratory settings. This enhancement is attributed to the hydrophobin coating the PET surface, thereby increasing its accessibility to enzymatic action [77,78]. Leveraging this beneficial property, Chen et al. [67] proposed that hydrophobin could be used to modify the surface hydrophobicity of PETase-displaying yeast cells. This modification would facilitate stronger attachment to PET substrates and enhance the degradation efficacy of the whole-cell biocatalyst system.

Chen et al. [67] developed a novel whole-cell biocatalyst in *Pichia pastoris* that mimics the natural two-stage PET degradation process. Their system incorporated *Trichoderma reesei* hydrophobin as a surface-anchored adhesion molecule, significantly boosting yeast cell binding to PET substrates. This modification proved particularly effective for degrading high-crystallinity PET (hcPET), with commercial PET bottles (45% crystallinity) showing conversion rates of 3.1% (Nestle), 3.1% (Coca-Cola), and 2.8% (Pepsi). These results represent a substantial improvement over the 0.01% degradation rate reported for native PETase by Yoshida et al. [53]. The biocatalyst demonstrated differential efficiency depending on PET crystallinity, achieving 3% conversion for hcPET versus 55% for low-crystallinity PET (lcPET) (Table 2). Remarkably, at 30 °C, the system showed a 328.8-fold increase in turnover rate for hcPET (45% crystallinity) compared to purified PETase [67].

### 5.3. Fungal Cutinase

Certain fungi, such as *Fusarium solanipisi*, exhibit significant cutinase activity [10,79]. The catalytic triad of this fungal cutinase (FsC) consists of Ser120, Asp175, and His188. The degradation mechanism begins when the oxygen from Ser120 attacks the carbonyl group of PET, forming a serine-terephthalate intermediate and an ether compound [79]. This enzymatic action ultimately cleaves PET into its constituent monomers: BHET, MHET and TPA. Subsequently, the ether compound’s oxygen forms a covalent bond with a hydrogen from the His188 residue, releasing EG. Finally, the oxygen of Ser120 retrieves a hydrogen from His188, releasing TA and regenerating the cutinase enzyme to initiate a new catalytic cycle [79].

To assess the plastic-degrading potential of a modified *Y. lipolytica* strain, Kosiorowska et al. [45] conducted shake-flask experiments. These cultures, grown for 144 h in YPD medium supplemented with 0.5 g of PCL film, were analyzed for film weight loss and the release of hydrolysis products like ε-caprolactone and 6-hydroxyhexanoic acid. The study found that strains overexpressing a cutinase from *F. solanipisi* achieved the greatest weight reduction, exceeding 90%. While strains expressing a cutinase or a lipase from *T. reesei* also degraded PCL, their weight loss was significantly lower at 9.8% and 15.8%, respectively. Co-expression of these two enzymes resulted in higher degradation than single-expression strains. Furthermore, the release of ε-caprolactone was positively correlated with the percentage of weight loss [45]. In related work, overexpression of the same *F. solanipisi* cutinase in the *Y. lipolytica* AJD strain was also shown to enhance the decomposition of PET film and the release of MHET and TPA [68] (Table 2 and Figure 3).

## 6. Future Directions

Most prior research using yeast models has focused on degrading a single plastic polymer, such as PET, PE, PU, PP or PCL. However, microplastics in natural environments constitute complex mixtures that can include PS, PP, PVC, polyvinyl alcohol (PVA), and many others, some of which are highly recalcitrant to biodegradation [3,80]. Consequently, there is a significant need to discover yeasts capable of degrading a broader spectrum of plastics, beyond those specialized for only PET or PE.

The exploration of plastic mineralization by soil and marine yeasts presents a promising frontier for addressing global plastic pollution. However, the realization of this potential is currently hampered by significant knowledge gaps. Firstly, the diversity of yeasts capable of participating in plastic mineralization remains vastly underexplored, with research heavily skewed towards a few yeast genera; thus, there is an urgent need to isolate and characterize novel yeast strains from plastic-polluted niches to build a repository of high-quality microbial resources. Secondly, the precise metabolic pathways and key enzymes responsible for mineralizing synthetic polymers in yeasts are poorly understood. Future research must, therefore, prioritize elucidating these molecular mechanisms through advanced genomics, transcriptomics, and proteomics. Unlocking the potential of these eukaryotic microbes will be pivotal in developing targeted and sustainable biotechnological solutions for plastic waste.

While extensive research has documented the role of yeasts in the biodeterioration of plastics—such as surface colonization, biofilm formation, and monomeric hydrolysis—the critical step of enzymatic depolymerization remains a significant bottleneck. A major challenge is the scarcity of yeast strains definitively proven to secrete active depolymerases (e.g., cutinases, lipases, esterases) capable of cleaving the backbone of synthetic polymers [16,17]. Most studies report weight loss or surface erosion without identifying the specific enzymes responsible. Therefore, a systematic screening of yeasts from diverse plastic-polluted environments—landfills, marine sediments, and recycling sites—is urgently needed to discover and characterize novel, high-performance strains with robust depolymerase activity.

Furthermore, the depolymerization process is highly substrate-specific. The enzymatic breakdown mechanisms for PET, PE, PU, PP and others differ fundamentally due to variations in chemical bonds (ester, urethane, carbon–carbon et al.) and polymer crystallinity [81]. Consequently, an enzyme highly efficient against one plastic type may show little to no activity against another. Future work must focus on elucidating the structure-activity relationship between depolymerases and different plastic substrates. This includes engineering enzyme active sites or developing enzyme cocktails tailored to degrade mixed plastic waste streams.

Oleaginous yeasts, characterized by their high lipase activity, are promising candidates for plastic degradation, with *Y. lipolytica* being a particularly well-studied example [82]. These yeasts are distributed across various clades and are commonly sourced from environments like soil, plants, and agricultural or industrial waste [82,83]. While this review highlights several other species, future research is likely to identify more highly efficient plastic-degrading yeasts, especially from plastic-contaminated marine and terrestrial habitats.

To date, genetic engineering for cell surface display has been applied primarily to *Y. lipolytica* [45,52,68,69], *Pichia pastoris* [67], and *C. tropicalis* [65]. Developing similar methods for other yeasts with inherent plastic-hydrolyzing capabilities remains a necessary future step. Although Zhang et al. [65] established a functional platform for a stable, long-term enzyme catalyst suitable for PET depolymerization on a large scale, their study was confined to a laboratory setting. Advancing this technology necessitates further investigation into the design of continuous, high-efficiency systems that can be scaled for industrial yeast operations.

Yeast cell surface display systems have employed bacterial PETase and fungal cutinase on separate platforms [45,52,65,67,68]. A novel approach would be to co-express *P. sakaiensis* PETase and *F. solani* cutinase within a single multi-enzyme display platform to investigate potential additive or synergistic effects.

The activity of PETase is known to be feedback-inhibited by the intermediate product MHET, making its synergy with MHETase crucial for efficient PET depolymerization [60,65]. Similarly, cutinase is also inhibited by MHET [84,85]. While the co-expression of cutinase with MHETase to alleviate this inhibition remains unexplored, an alternative strategy has been demonstrated. Fusion of an elastin-like polypeptides (ELPs) tag to cutinase altered its product profile, yielding only TPA instead of the mixture of TPA and MHET produced by the wild-type enzyme. This presents a simplified method for mitigating intermediate inhibition compared to existing techniques [85].

The speed at which a polymer breaks down is influenced by multiple elements, such as its chemical composition, molecular weight, and level of crystallinity [81]. These large molecules contain organized crystalline zones and disorganized amorphous zones; the amorphous regions are responsible for imparting flexibility [81]. Polymers with very high crystallinity, such as PE (up to 95%), tend to be stiff and have poor impact resistance. For instance, PET plastics have a crystallinity of 30–50%, a key factor contributing to their slow microbial breakdown. It is estimated that complete degradation in natural settings could exceed 50 years, extending to hundreds of years in ocean environments where temperatures and oxygen levels are lower [81].

Enzymatic degradation typically proceeds in two phases: first, enzymes adsorb onto the polymer surface, and then they catalyze the hydrolysis of its bonds. One strategy to improve this process involves using hydrophobin to alter the surface hydrophobicity of yeast cells engineered to display PETase, thereby promoting their attachment to hydrophobic PET and increasing the efficiency of the whole-cell biocatalyst [67]. However, the combined expression of fungal cutinase with hydrophobin has not yet been explored. To date, even the most effective co-display systems have achieved only limited degradation—approximately 3% over 18 h at 30 °C—for highly crystalline PET, such as that found in bottles with 45% crystallinity [67]. This raises the question of whether a synergistic system incorporating PETase, MHETase, cutinase, and hydrophobin could achieve significantly higher degradation efficiency.

## 7. Conclusions

Over the past few decades, yeasts have drawn growing interest as promising candidates for plastic biodegradation, owing to their fast growth, broad metabolic capabilities, and genetic amenability. High levels of plastic mineralization and depolymerization efficiency have been observed in yeasts. The ester linkages that connect monomer units within plastics are susceptible to cleavage by yeast-derived enzymes, including esterases, lipases, and cutinases. While yeast models for the degradation of PET and PE have been extensively characterized, further exploration is still required to identify yeast species that can break down alternative plastic types or a wider range of plastic materials. To date, genetically engineered cell surface display systems have demonstrated significantly improved degradation efficiencies when applied to low-crystallinity plastics. Nevertheless, yeast engineering strategies for breaking down polymers with very high crystallinity remain underdeveloped; for instance, the combined expression of fungal cutinase alongside hydrophobin represents one such approach that warrants further investigation.

## Figures and Tables

**Table 1:** Identified plastic degradative yeasts.

Species (Class)	Enzymes	Pretreatments	Efficiency (Plastic Type)	Ref.
*Moesziomyces antarcticus* (Basidiomycetes)	Lipase	PET bottle film was cut into squares of 0.5 cm and 0.1 mm thickness	**PET** depolymerization	[24]
*Moesziomyces antarcticus*	Lipase	Cut into squares of 0.5 cm and 0.1 mm thickness	0.4% pellet and bottle **PET** after 3 weeks	[25]
*Yarrowia lipolytica* IMUFRJ 50682 (Saccharomycetes)	Lipase	Materials were ground into particles < 1.18 mm	Using post-consumer **PET** as a carbon source	[26]
*Candida tropicalis* (Saccharomycetes)	Lipase, Esterase, PEG dehydrogenase	Grounded into microplastic pieces	50% **PET**-based microplastics after 30 days	[27]
*Vanrija*sp. SlgEBL5 (Saccharomycetes)	Lipase, Esterase	Micronized into granules of 300 μm; radiated with UV for 15 min	10% 75–300 μm **microPETs** after 30 days	[28]
*Rhodotorula mucilaginosa* (Microbotryomycetes)	Not studied	UV A/B dose corresponding to ~50 and ~125 days of UV irradiation	3.8% **PE** mineralization per year in the marine environment	[22]
*Debaryomyces hansenii* RELF8 (Saccharomycetes)	Lipase, Cutinase	Cut into 5 × 5 cm^2^ pieces in film format	2.5–5.5% linear low-density **PE** after 30 days	[29]
*Meyerozyma carpophila* M6.0.2 (Saccharomycetes)	Laccase, Peroxidase, Cytochrome P450, Esterase, Lipase	Low-density PE granules (melt index: 190 °C/2.16 kg); exposed to UV light for 1 h	0.5% low-density **PE** granules after 10 days	[30]
*Exophilia* sp. NS-7 (Saccharomycetes); *Rhodotorula* sp. NS-12	Not studied	PU films were cut into squares of 2 × 2 cm^2^	**PPU** mineralization	[21]
*Sakaguchia* sp. BIT-D3 (Cystobasidiomycetes)	Cutinase	Plastic pellets were dissolved at 60 °C to produce a plastic emulsion	**PPU**, **PCL** and **PBS** depolymerization	[31]
*Diutina rugosa* (Saccharomycetes)	Lipase	Micronized into 0.2 μm particles; rigid PU foam was heated at 350 °C for 20 min	**PU** deterioration after pyrolysis	[32,33]
*Candida ethanolica*	Not studied	PU fabrics were melted at 180 °C at 8 bar pressure, and then cut into 4–5 cm	**PU** deterioration 45–50 °C	[34]
*Hortaea werneckii* M-3 (Dothideomycetes)	Cutinase, Urease	Grounded into microplastic pieces	Utilizing **PU** as a sole carbon source	[35]
*Candida*, *Meyerozyma*, *Rhodotorula*, *Malassezia* (Basidiomycetes), *Rhodosporidiobolus* (Microbotryomycetes)	Not studied	Not mentioned	**PU**, **PE**, **PE**, **PVC**, **PET** deterioration respectively	[36]
*Yarrowia lipolytica*	Not studied	PP pellets were heated at 540 °C for 195min	**PP** upcycling process after pyrolysis	[37]
*Rhodotorula dairenensis* EXF-13500; *Rhodotorula* sp. EXF-10630; *Wickerhamomyces anomalus* EXF-6848 (Saccharomycetes)	Not studied	Plastic films were cut into 1 cm^2^ pieces and then sterilized by 96% ethanol	**PP**,low-density **PE**, **PET** deterioration respectively	[38]
*Rhodotorula*	Not studied	Foam plastics without pretreatment	**PP**, **PU** and **EVA** deterioration	[39]
*Malassezia*	Not studied	Polymer emulsion was prepared	**PVC**depolymerization	[40]
*Buckleyzyma aurantiaca* (Cystobasidiomycetes); *Kluyveromyces* spp. (Saccharomycetes)	Not studied	0.5 mm thick plasticized PVC sheets were formulated	**PVC** deterioration	[41]
*Diutina rugosa*; *Candida cylindracea*	Lipase	Highly sensitive film was prepared	**P(BS-co-HS)** deterioration	[42]
*Moesziomyces antarcticus*	Not studied	Plastic film without pretreatment	**PBSA** mineralization in 55 days	[23]
*Cryptococcus* sp. (Tremellomycetes)	Cutinase	Plastic emulsion was prepared	Hydrolysis of **PLA**, **PBS**, **PCL** and **PHB**	[43]
*Cryptococcus* sp.	Lipase	Heated at 55 °C/140 °C for PBSA/PBS for 7 min to produce plastic films	Hydrolysis of **PBS** and **PBSA**	[44]
*Yarrowia lipolytica* AJD	Esterase, Lipase	Plastic emulsion was prepared	1% **PCL** film after 144 h	[45]
*Candida* sp.	Lipase	Films were obtained by molding at 120 °C for PCL and 170 °C for PBSA	Low molecular weight **PCL** and **PBSA** deterioration	[46]

**Table 2:** Yeast cell surface display for plastic degradation.

Species (Strain)	Enzymes Displayed	Efficiency	Reference
*Yarrowia lipolytica* AJD	Overexpression of cutinase from *Trichoderma reesei*	9.8% PCL film after 144 h	[45]
*Yarrowia lipolytica* AJD	Overexpression of *Y. lipolytica* lipase and *T. reesei*cutinase	15.8% PCL film after 144 h	[45]
*Yarrowia lipolytica* AJD	Overexpression of *Fusarium solanipisi* cutinase with or without *Y. lipolytica* lipase	>90% PCL film after 144 h	[45]
*Yarrowia lipolytica* AJD	Surface display of PETase from *Piscinibacter sakaiensis*	44.74% PET film at 28 °C 240 h	[52]
*Candida tropicalis* CU-208	Surface display of PETase and MHETase from *P. sakaiensis*	20 g PET powder (13.84 % crystallinity) completely degraded at 45 °C in 7 d (5 L)	[65]
*Yarrowia lipolytica* Po1f	Surface display of PETase from *Piscinibacter sakaiensis*	Enhanced TPA and EG releasing	[66]
*Pichia pastoris* GS115	Surface codisplay of PETase from *P. sakaiensis* and hydrophobin from *T. reesei*	3% and 55% for hcPET (45% crystallinity) and lcPET (6.3% crystallinity) at 30 °C 18 h	[67]
*Yarrowia lipolytica* AJD	Overexpression of its lipase and two extracellular proteases	Enhanced MHET and TPA releasing and PET film decomposition	[68]
*Yarrowia lipolytica* AJD	Overexpression of *F. solanipisi* cutinase and *Y. lipolytica* lipase	Enhanced MHET and TPA releasing and PET film decomposition	[68]
*Yarrowia lipolytica* AJD	Overexpression of its lipase and two extracellular proteases	2% PCL film after 144 h	[45,69]

## Data Availability

No new data were created or analyzed in this study. Data sharing is not applicable to this article.
